# Effects of a comprehensive menstrual and sexual reproductive health intervention on RTI symptoms among adolescent girls in the Mwanza region: a mixed-methods analysis

**DOI:** 10.1186/s12978-025-02087-x

**Published:** 2025-11-12

**Authors:** Onike Mcharo, Anthony Kapesa, Jacqueline Materu, John Luwayi, Fauzia Nahay, Namanya Basinda, Elieza Chibwe, Elialilia Okello

**Affiliations:** 1 Catholic University of Health and Allied Science, Mwanza, Tanzania; 2 Mwanza Intervention Trials Unit, National Institute for Medical Research, Mwanza, Tanzania; 3 National Institute for Medical Research, Mwanza, Tanzania

**Keywords:** Reproductive tract infections, Menstrual hygiene practices, School-based comprehensive reproductive health intervention

## Abstract

**Background:**

Poor menstrual hygiene management (MHM) has been implicated in increasing RTI symptoms. The Partnering to Support Schools to Promote Good Menstrual Health and Well-being (PASS MHW) intervention was designed to address menstrual-related challenges among schoolgirls. However, no comprehensive analysis has evaluated its impact on menstrual management practices or its potential influence on reducing reproductive tract infection (RTI) symptoms.

**Objective:**

This study examined the effects of a comprehensive Menstrual and Sexual Reproductive Health (MSRH) intervention on reproductive tract infection (RTI) symptoms among adolescent girls and aimed to identify the key predictors of these changes.

**Methodology:**

This was a mixed-methods secondary analysis of the PASS MHW project that involved surveys from 424 secondary school girls to assess changes in RTI symptoms and menstrual hygiene practices. In addition, 20 in-depth interviews were conducted with subsamples of girls referred for RTI treatment to assess their experience with health care services.

**Results:**

There was a significant postintervention increase in the proportion of schoolgirls using hygienic menstrual products, increasing from 32 to 77% (*p* = 0.001). RTI symptoms presented mixed outcomes: thick, white, clumped discharge decreased from 33 to 26% (*p* = 0.026), while fish-smelling discharge increased from 6 to 16% (*p* = 0.001), with urban participants experiencing a significant increase (OR = 2.15, 95% CI: 1.31–3.52, *p* = 0.002. Among older adolescents, there was an increase in genital symptoms, including itching, sores, and rash, particularly redness or irritation, although adjusted analysis indicated a lower likelihood compared to younger adolescent (OR = 0.67, 95% CI: 0.46–0.98, *p* = 0.039). Referral services encounter barriers, including poor service quality, financial limitations, stigma, and issues related to obtaining consent.

**Conclusion:**

The PASS MHW intervention improved the use of hygienic menstrual products but had mixed effects on RTI symptoms. Some symptoms decreased, whereas others increased, especially among urban participants and younger adolescents. Barriers in referral services, including quality, cost, stigma, and consent issues, remain challenges.

## Introduction

Reproductive health is a critical public health concern, particularly for adolescent girls, as reproductive tract infections (RTIs) pose significant health challenges worldwide [1]. Despite their serious impact, RTIs are often underdiagnosed and inadequately treated, particularly in low- and middle-income countries (LMICs), where access to healthcare services is limited [2–5]. One major risk factor contributing to RTIs among adolescent girls is poor menstrual hygiene management (MHM) [6–8]. Practices such as the use of unhygienic materials; limited access to water, sanitation, and hygiene (WASH) facilities; and inadequate menstrual education increase the vulnerability of adolescent girls to these infections [8–11]. This vulnerability can lead to severe long-term health consequences, including infertility, reproductive organ damage, and increased susceptibility to HIV [12–16].

Studies have identified several contributing factors to poor MHM practices and the prevalence of RTIs, including rural–urban disparities, inadequate WASH infrastructure, and limited access to sanitary menstrual products [17, 18]. Studies from low resource countries and from sub-Saharan Africa suggest that adolescent girls using unhygienic materials, such as cloths, are at increased risk of RTIs symptoms owing to prolonged exposure to harmful bacteria and pathogens [19–23]. Moreover, insufficient WASH facilities hinder the ability to change menstrual products regularly and maintain proper hygiene, further compounding the risk of infections [9, 24–26]. However, many existing studies focus on isolated factors and do not address the combined impact of comprehensive reproductive health interventions. This gap limits our understanding of how integrated approaches can mitigate these challenges and improve health outcomes for adolescent girls.

Previous studies have explored different aspects of MHM and its connection to RTIs, but the complex relationships among education, access to menstrual products, WASH infrastructure, and healthcare access have not been fully examined. Filling these gaps is essential for designing effective, targeted interventions that improve reproductive health outcomes and the overall well-being of adolescent girls. Understanding these interdependencies is crucial for developing integrated strategies that can address the multifaceted nature of reproductive health issues in this population.

The recently completed comprehensive menstrual and sexual reproductive health (MSRH) intervention named the Partnering to Support Schools to Promote Good Menstrual Health and Well-Being (PASS MHW) implemented by the Mwanza Intervention Trial has shown its potential to improve menstrual hygiene practices, increase health service utilization, and empower adolescents with the knowledge and resources needed to make informed decisions on their reproductive health [27, 28]. However, significant gaps remain in understanding how this intervention influences the prevalence and patterns of RTIs symptoms, particularly among different demographic groups and settings. This study seeks to fill this gap by assessing the effects of this intervention on RTIs symptoms among adolescent girls. Unlike previous studies, this study also investigated the predictors of RTIs prevalence and explored adolescent girls'perceptions of barriers to healthcare access and utilization. The findings provide actionable insights that will strengthen reproductive health interventions for adolescent girls in LMICs and highlight the importance of integrating education, access to menstrual products, and improved WASH and healthcare services to improve the reproductive health of adolescent girls.

## Methods

### Study setting, design and population

This study employed a mixed-methods secondary data analysis, drawing on data from the PASS Menstrual Health and Wellbeing (MHW) project in Mwanza, Tanzania [27]. The aim was to assess the impact of comprehensive menstrual and sexual reproductive health (MSRH) interventions on the prevalence of self-reported reproductive tract infection (RTI) symptoms and to identify predictors of change among adolescent girls in Mwanza. The intervention was implemented in four secondary schools, two located in urban areas and two in rural settings, selected through a multistage sampling process. Survey data were collected from all adolescent girls who were present for both the baseline (the start of intervention) and endline (12-month post intervention) assessments. The intervention had five key components: i) Comprehensive MSRH education, which consisted of a total of 10-h student education sessions delivered over 5 days delivered to boys and girls. The sessions covered the biology and social aspects of menstruation; reproductive health [urinary tract infections (UTIs), reproductive tract infections (RTIs), sexually transmitted infections (STIs) symptoms], pregnancy and pregnancy prevention, and gender issues including healthy relationships and gender-based violence (GBV). The five-day sessions were followed by two “booster” sessions in weeks six and twelve, during which facilitators returned to the schools for question-and-answer short sessions with students to clarify issues. ii) Provision of menstrual management kits which contained two reusable menstrual products: a menstrual cup and a pack of washable pads, two pairs of underwear, a bar of soap. iii) In addition to the pain management sessions delivered as part of the education sessions, one teacher at each school was selected and given additional training on pain management and dispensing of analgesic drugs. Using the existing essential medicine procurement system, each school was supplied with two conventional over-the-counter analgesics. iv) Minor WASH improvements were implemented in each school. The improvements were guided by school needs and involved repairing toilets and/or menstrual rooms, providing locks on doors, soap, and buckets, and improving access to water in the toilets and/or menstrual rooms. v) The final component was stakeholder engagement involving teachers, parents, and local government leadership. A more detailed description of the intervention has been provided in the previous publications [28, 29].

The study population comprised adolescent girls aged 13–20 years who were in their second and third years of secondary school at the time of enrollment. A total of 424 girls participated in the baseline survey, with 408 completing the follow-up assessment at endline, 12 months after the intervention. The quantitative component specifically focused on those who started menstruating and attended reproductive health (MSRH) education sessions. The RTI symptoms were defined as the self-reported experience of lower genital tract infection-related signs in their last menstrual period and measured with eight questions asking if girls have experienced any of the symptoms such as abnormal vaginal discharge, genital pain, General itching or burning sensation in genital area, burning feeling with urination, Sores, blisters, or ulcers in the genital area or inside vagina and rash or redness in genital area. These questions have 2 responses (No, I haven’t experienced = 0 and yes, I have experienced = 1). Menstrual product used in the last period were assessed by 1 question this will be assessed by answering ‘yes’ if they used the product mentioned and ‘no’ if they didn’t use the product mentioned and multiple responses were allowed meaning those that used more than one product mentioned. The menstrual product were grouped into to two categories which were hygienic product (AFRI pads or other re-usable pads, Always or other pads you can throw away, locally made pads you throw away, Saalt Cup or other menstrual cup) and unhygienic product which were (fabric, clothes-e.g. face towels, hankies), socks, toilet paper/newspaper, knickers only and cotton wool). Menstrual product use was assessed by asking whether the respondent used specific items during their last period. Multiple responses were allowed. Products were categorized as hygienic (e.g., commercial disposable pads, reusable pads, menstrual cups) or unhygienic (e.g., cloth, tissue, cotton wool, underwear alone).

During the implementation of the PASS project, the MSRH education session introduced students to menstrual and sexual reproductive health (MSRH), including symptoms, treatments and management of various urogenital infections and its impact of the reproductive organs. The MSRH education also encouraged students to seek help if they experienced any concerning symptoms. This led to 120 girls (25%) reaching out to the research team, of whom 41 were referred to local health facilities for further evaluation and treatment. To better understand these experiences, the study included a qualitative component, involving in-depth interviews with 20 girls who had reported more severe symptoms during the sessions.

### Data analysis

Data was extracted and analyzed via STATA version 17 for quantitative analysis, and themes were identified via NVivo software. Quantitative data were extracted and analyzed, and descriptive statistics were used to summarize the data, with frequencies and percentages for categorical variables and means and standard deviations for continuous variables. The generalized estimating equation (GEE) model accounts for the within subject correlation between repeated measurements from the same individual, and model the outcome variable of RTI symptoms, considering potential confounding factors that remain consistent over time. To measure reproductive tract infection (RTI) symptoms, girls were asked eight yes/no questions regarding specific symptoms, such as abnormal discharge, genital itching or pain, intermenstrual bleeding, painful urination, sores or blisters, and redness or rash in the genital area. Responses were binary (0 = No, 1 = Yes), allowing the creation of a symptom prevalence score. Demographic variables included in the model such as age, location (urban/rural), and school grade were selected based on their relevance in existing literature and their potential influence on menstrual health and RTI risk. These variables were chosen as a priori to explore disparities in RTI symptom prevalence and to identify subgroups that may benefit most from targeted interventions.

Menstrual product use was assessed by asking whether the respondent used specific items during their last period. Multiple responses were allowed. Products were categorized as hygienic (e.g., commercial disposable pads, reusable pads, menstrual cups) or unhygienic (e.g., cloth, tissue, cotton wool, underwear alone). Comparisons of menstrual management practices between baseline and endline were conducted to assess changes over time. Frequencies and percentages were calculated to examine the proportional changes in menstrual hygiene practices across the two time points, providing insight into the effectiveness of the intervention. To assess the association between menstrual practices and related RTI symptoms at both at baseline and endline, Chi-square tests were employed. This statistical test is appropriate for examining relationships between categorical variables in this case, menstrual hygiene practices (explanatory variables) and self-reported RTI symptoms (outcome variables) at different time points.

Qualitative data were collected in Kiswahili, the main language used in Tanzania. The audio-recorded interviews were transcribed verbatim and translated and translated int English by the first author who is fluent in both Kiswahili and English. Analysis started with two authors reading through a sample of transcripts each. They independently developed draft codes using a combination of both priori and grounded codes and met to discuss and harmonize the codes. The first author used the final code book to code and retrieve segments of the data. After coding all the data, similar codes were grouped under themes. Memos describing the patterns and variations in the different segments of retrieved data were written. An inductive approach was used to provide new insights and richer understanding of the data without using preconceived categories. Verbatim quotations from the data were used to highlight key study findings.

## Results

### Background characteristics of the study participants

The study included adolescent schoolgirls from both rural and urban areas, highlighting differences in access to resources, menstrual product use, and sociodemographic characteristics. The participants were categorized into young adolescents (13–16 years) and older adolescents (17–20 years), with young adolescents comprising 78% of the study sample and 68% of the participants attending urban schools. Menstrual product use during the last menstrual period showed a notable shift, with the use of hygienic products increasing from 32% (*n* = 125) at baseline to 77% (n = 302) at endline (12-month post intervention), whereas the use of unhygienic products decreased from 68% (264) to 23% (89). A summary of participants’ sociodemographic characteristics and product use among schoolgirls over the study period is provided below (Table 1).

**Table 1:** Sociodemographic characteristics of the participants from Comprehensive Menstrual and Sexual Reproductive Health Intervention on RTI Symptoms among Adolescent Girls in the Mwanza Region

Participants characteristics	Baseline *N* = 424	Endline *N* = 408
	**n (%)**	**n (%)**
Age group		
Young adolescent 13–16	330(78)	173(42)
Older adolescent 17–20	93(22)	235(58)
Location		
Urban (School 1 & 4)	271 (64)	271(66)
Rural (School 2 & 3)	153 (36)	137(34)
Menstrual product use during last menstrual period(n = 391)		
Hygienic Products	125 (32%)	302 (77)
Unhygienic products	264 (68)	89 (23)

### Changes in the prevalence of reported RTI symptoms among adolescent schoolgirls

Our study shows significant changes in the prevalence of reproductive tract infection (RTIs) among participants between baseline and endline, with variations by age group and location. A decrease in vaginal discharge with a bad smell was observed among older adolescents, with the prevalence decreasing from 74 to 32%, a 42% reduction (OR = 0.56, 95% CI: 0.34–0.92; p = 0.023). In contrast, urban participants experienced an increase in this symptom, increasing from 37 to 53% (OR = 2.15, 95% CI: 1.31–3.52, p = 0.002), whereas rural participants experienced a reduction from 63 to 47%.

For green or yellow vaginal discharge, divergent trends emerged with age. The prevalence decreased substantially among older adolescents, from 65 to 15%, whereas that among younger adolescents increased, from 35 to 75%, older adolescents had lower odds of experiencing the symptom (OR = 0.53, 95% CI: 0.28–1.02, *p* = 0.058) compared to younger adolescents, (OR = 0.67, 95% CI: 0.46–0.98, p = 0.039) suggesting a potential protective effect of age. Additionally, rural participants had a significantly greater probability of experiencing this symptom than urban participants did (OR = 2.74, 95% CI: 1.43–5.22, *p* = 0.002).

Other RTI symptoms showed minimal variation without statistical significance. The proportion of participants reporting a burning sensation while urinating increased slightly from 22% (n = 95) to 24% (n = 99), but this change was not statistically significant (*p* = 0.662). Similarly, the prevalence of general itching or burning in the genital area remained constant at 28% (n = 119) (p = 0.790). The occurrence of sores, blisters, or ulcers in the genital area decreased slightly from 18% (*n* = 76) to 15% (*n* = 62), but this reduction was not statistically significant (*p* = 0.273). Similarly, the prevalence of rash or redness in the genital area increased slightly from 17% (n = 70) to 19% (n = 77), which also did not reach statistical significance (*p* = 0.532) (Table 2)

**Table 2:** Changes in the prevalence of reported RTI symptoms among adolescent schoolgirls from Comprehensive Menstrual and Sexual Reproductive Health Intervention on RTI Symptoms among Adolescent Girls in the Mwanza Region

RTI Symptoms	Baseline n (%)	Endline n (%)	*P*- Value
Vaginal discharge that is green or yellow			
Experienced	20(5)	20(5)	1.000
Not experienced	403(95)	388(95)	
Vaginal discharge that is thick white and clumpy			
Experienced	138(33)	105(26)	0.026
Not experienced	285(67)	303(74)	
Vaginal discharge that has a strong, bad smell (fishy smell)			
Experienced	28(6)	48(16)	0.001
Not Experienced	395(94)	360(88)	
Burning sensation while urinating			
Experienced	95(22)	99(24)	0.662
Not experienced	328(78)	309(76)	
General itching or burning sensational in your genital area			
Experienced	119(28)	119(29)	0.790
Not Experienced	304(72)	289(71)	
Sores, blisters or ulcers in the genital area or inside vaginal			
Experienced	76(18)	62(15)	0.273
Not Experienced	347(82)	346(85)	
Rash or redness in the genital area			
Experienced	70(17)	77(19)	0.532
Not experienced	353(83)	331(81)	

### Changes in reported RTI symptoms by age group and area of residence (rural/urban)

Our study revealed significant changes in the prevalence of reproductive tract infection (RTIs) symptoms among participants between baseline and endline, with variations by age group and location. A decrease in vaginal discharge with a bad smell was observed among older adolescents, with the prevalence decreasing from 74 to 32%, a 42% reduction (OR = 0.56, 95% CI: 0.34–0.92; *p* = 0.023). In contrast, urban participants experienced an increase in this symptom, increasing from 37 to 53% (OR = 2.15, 95% CI: 1.31–3.52, p = 0.002), whereas rural participants experienced a reduction from 63 to 47%. For green or yellow vaginal discharge, divergent trends emerged with age. The prevalence decreased substantially among older adolescents, from 65 to 15%, whereas that among younger adolescents increased, from 35 to 75%, while the raw percentages suggest a worsening among younger adolescents and improvement among older ones, the adjusted analysis confirms that older adolescents had lower odds of experiencing the outcome compared to their younger counterparts (OR = 0.53, 95% CI: 0.28–1.02, *p* = 0.058). This suggests that despite the apparent increase in unadjusted prevalence among younger adolescents, the adjusted results support a protective effect of age. Additionally, rural participants had a significantly greater probability of experiencing this symptom than urban participants did (OR = 2.74, 95% CI: 1.43–5.22, *p* = 0.002).

The prevalence of burning sensation while urinating was 26% lower among older adolescents (OR = 0.64, 95% CI: 0.46–0.90, p = 0.010), whereas the prevalence among urban participants increased from 46 to 62% (OR = 1.93, 95% CI: 1.37–2.70, p < 0.001). Similarly, general itching or burning sensations increased among younger adolescents (27% to 63%) but decreased among older adolescents (73% to 37%, OR = 0.73, 95% CI: 0.53–1.00, p = 0.054). Urban participants were more likely to experience this symptom (OR = 1.67, 95% CI: 1.21–2.30, *p* = 0.002).

Sores, blisters, or ulcers decreased among older adolescents but increased among younger adolescents, although no significant associations with age or location were found. The prevalence of thick white, clumpy discharge decreased among older adolescents but increased in urban areas, with a 21% increase in urban participants (OR = 1.39, 95% CI: 1.02–1.90, *p* = 0.034). The prevalence of rash or redness in the genital area increased significantly among younger adolescents, rising from 27% at baseline to 66% at endline. In contrast, older adolescents were less likely to report this symptom. This age-related difference was statistically significant (OR = 0.67, 95% CI: 0.46–0.98; p = 0.039), meaning that older adolescents had 33% lower odds of reporting rash or redness in the genital area compared to their younger counterparts. This suggests a higher burden of symptoms among younger adolescents over time. Although urban participants had slightly higher prevalence rates than did rural participants, the difference was not statistically significant (OR = 1.25, 95% CI: 0.87–1.80, *p* = 0.212) (Table 3).

**Table 3:** Present changes in reported RTI symptoms by age group and area of residence (rural/urban) from Comprehensive Menstrual and Sexual Reproductive Health Intervention on RTI Symptoms among Adolescent Girls in the Mwanza Region

		**Baseline** **n (%)**		**Endline** **n (%)**			
		**No Symptoms**	**With symptoms**	**No Symptoms**	**With symptoms**	**OR (95%CI)**	***P* Value**
Vaginal discharge with a bad smell							
Age Group	Young adolescent	310 (79)	7(26)	157 (44)	32 (68)	0.56(0.34, 0.92)	0.023
	Older adolescent	85 (21)	20(74)	203(56)	15 (32)		
Location	Urban	261(66)	10(37)	245(68)	25 (53)	2.15(1.31, 3.52)	0.002
	Rural	134(34)	17(63)	115 (32)	23 (47)		
Green or yellow vaginal discharge							
Age Group	Young adolescent	86 (21.3)	7(35)	168 (43)	15 (75)	0.53(0.28, 1.02)	0.058
	Older adolescent	317(78.7)	13(65)	220 (58)	5 (15)		
Location	Urban	263(65)	8(40)	263(68)	8 (40)	2.74(1.43, 5.22)	0.002
	Rural	140(35)	12(60)	125(32)	12 (60)		
Burning sensation while urinating							
Age Group	Young adolescent	61 (19)	32(34)	134 (43)	60 (61)	0.64(0.46, 0.90)	0.010
	Older adolescent	267 (81)	63(66)	175 (57)	39 (40)		
Location	Urban	227(69)	44(46)	210(68)	61 (62)	1.93(1.37, 2.70)	0.000
	Rural	101(31)	51(54)	99(32)	38 (38)		
General itching or burning sensation							
Age Group	Young adolescent	61 (20)	32(27)	160 (55)	75 (63)	0.73(0.53, 1.00)	0.054
	Older adolescent	243 (80)	87(73)	129 (45)	44 (37)		
Location	Urban	203 (67)	68(57)	204(71)	67 (56)	1.67(1.21, 2.30)	0.002
	Rural	101 (33)	51(43)	85(29)	52 (44)		
Sores, blisters, or ulcers							
Age Group	Young adolescent	78 (22)	15(20)	154 (45)	43 (69)	0.77(0.52, 1.13)	0.183
	Older adolescent	269 (78)	61(80)	196(55)	19 (31)		
Location	Urban	222 (64)	49(64)	230(66)	41 (66)	1.12(0.77, 1.64)	0.533
	Rural	125 (36)	27(36)	116(34)	21 (34)		
Thick white, clumpy vaginal discharge							
Age Group	Young adolescent	63 (22)	15(20)	133 (44)	43 (69)	1.04(0.76, 1.43)	0.767
	Older adolescent	222 (78)	61(80)	170 (56)	19 (31)		
Location	Urban	190 (67)	81(59)	205(68)	84 (80)	1.39(1.02, 1.90)	0.034
	Rural	95 (33)	57(41)	98(32)	21 (20)		
Rash or redness in genital area							
Age Group	Young adolescent	74 (21)	19(27)	147 (44)	1 (66)	0.67(0.46, 0.98)	0.039
	Older adolescent	279 (79)	51(73)	184 (56)	26 (34)		
Location	Urban	230 (65)	41(59)	221(67)	38(65)	1.25(0.87, 1.80)	0.212
	Rural	123 (35)	29(43)	110(33)	27 (35)		

### Barriers faced by adolescent girls in accessing RTIs treatment and strategies for improvement

During the intervention, 435 out of 498 adolescent girls attended the third day of the MSRH education sessions, which focused on physical health topics including urogenital infections such as RTIs, STIs, and UTIs. The sessions addressed the prevalence, symptoms, diagnosis, and treatment of these infections, while emphasizing the importance of timely health-seeking, particularly important given the common barriers adolescents face, such as stigma, fear of parental judgment, and lack of financial resources. Of the attendees, 273 were from urban schools and 162 from rural schools. Six weeks after the education sessions, facilitators returned to schools for booster visits, during which 120 girls (78 from urban schools and 42 from rural schools) came forward reporting persistent or severe symptoms. Although clinical diagnosis and treatment were not part of the original intervention design, 41 girls (25 urban, 16 rural) were ethically referred to nearby health facilities for evaluation and care. At endline, 20 of the referred girls were purposively selected for in-depth interviews to explore their experiences accessing health services, the care received, and ongoing challenges hence including these experiences in the analysis is both ethically and methodologically justified.

The increase in self-reported symptoms and care-seeking behavior reflects the impact of the intervention in raising awareness and empowering students to act on their health needs. Excluding these outcomes would overlook a critical effect of the education sessions. The qualitative findings further enriched the results by providing insight into the real-life challenges adolescents face in navigating healthcare systems such as cultural stigma, dismissive provider attitudes, and logistical barriers. These accounts reinforced the urgent need for school-linked, adolescent-friendly services and the integration of health education with accessible care pathways. By including these voices, the study offers a more comprehensive understanding of how such interventions can drive both awareness and action among adolescents and provides practical recommendations for improving reproductive health programming in similar contexts.

The results from the interview show that adolescent girls were generally willing to seek RTIs treatment when supported by a well-structured referral system, positive healthcare experiences, and accessible services. However, barriers such as long wait times, medication shortages, financial constraints, and insufficient follow-up care often compromise the effectiveness of treatment. Furthermore, many girls face social‒cultural barriers and family dynamics, resulting in stigma, misinformation, and fears, which discourage adolescents from seeking care.

Multiple challenges within healthcare facilities hinder adolescent girls’ access to timely and adequate reproductive health services. These challenges persist across both rural and urban settings, affecting the overall effectiveness of healthcare services. In urban areas, long wait times have emerged as a significant barrier, whereas both urban and rural health facilities frequently experience shortages of essential medications, impacting treatment outcomes. As a result, some girls received only partial care due to these systemic inconsistencies. As one rural participant described,

*"When I went to the health facility, the healthcare providers were few, and the medication some of us needed was not available."* (Female, 17 years, Rural).

Family dynamics play a critical role in shaping healthcare-seeking behaviors and follow-up care among adolescent girls. Maternal support often facilitates access to treatment, with some girls relying on their mothers for guidance and assistance in seeking medical care. However, for others, concerns about stigma, privacy, or family attitudes toward reproductive health problems led to reluctance to disclose symptoms or pursue care. These dynamics significantly influenced not only the decision to seek initial treatment but also adherence to follow-up care. Additionally, dissatisfaction with their initial healthcare experience or structural barriers can impact adherence to follow-up care. As one participant expressed her hesitancy to return,

*"No, I didn’t go for a follow-up because the medication I received did not help much. The symptoms have not gone away; they are still the same, and I could not go back to the hospital because I felt the medicine did not help."* (Female, 16 years, urban).

In addition, financial constraints also pose a significant barrier, with some adolescents being unable to afford consultation fees, medications, or treatments. One participant noted,

*"If you don’t have money, you’ll just be examined and told what issues you have, but you will need to buy the medication yourself."* (Female, 17 years, Rural).

Sociocultural factors also play a critical role in limiting adolescent girls'access to RTI care. These societal norms and misconceptions created an environment of silence and shame around reproductive health issues, further preventing adolescents from seeking necessary medical care. Many girls reported feeling embarrassed or ashamed to seek care for reproductive health problems because of fears of judgment from healthcare providers and/or their communities. One participant shared,

*"Currently, there is no stigma when you go for services, but many feel embarrassed. When you get there, the doctor might ask you to undress, which makes you feel shy. Additionally, explaining that you have sexually transmitted infections causes embarrassment, so many girls avoid treatment."* (Female, 14 years, urban).

The requirement for parental or teacher permission to visit health facilities was another significant barrier, as adolescents often hesitate to disclose reproductive health concerns due to fear of being misunderstood or judged. A participant noted,

*"If you seek permission to go to the hospital, you might be asked what you are suffering from. A girl might find it challenging to explain why she is going for a check-up or what she is experiencing, so questions from teachers or parents can be a barrier to seeking medical care. This is easier with other illnesses such as typhoid or malaria, but with reproductive health issues, you might feel embarrassed to tell your parents."* (Female, 17 years, urban).

Societal norms and misconceptions created a culture of silence and shame, particularly regarding sexually transmitted infections (STIs), RTIs, and HIV/AIDS. Fear of being labeled promiscuous or facing social ostracism prevented many girls from seeking necessary medical care. One participant shared,

*"The obstacles that girls face can come from their parents, where a parent might tell them that the illness is their fault and stigmatize them, which hurts the girl and makes her lose the freedom to go to a health center out of fear that the parent will think badly of her, assuming she is promiscuous, which is why she has these diseases."(Female, 15 years, urban).*

## Suggestions for tailored Sexual and Reproductive Health (SRH) interventions

The participants emphasized a multifaceted approach to improving adolescent girls'access to reproductive health services, with a particular focus on the role of school-based interventions. The intervention’s educational component was highlighted as critical in addressing RTIs by improving awareness and promoting preventive practices. Education about hygiene, early detection, and safe practices empowered girls to seek timely treatment and reduce infection rates. As one participant explained,

*"Through screening, a person can determine whether they are safe or not. Education helps girls understand how infections are transmitted and how to maintain hygiene to prevent reproductive infections."(Female, 16 years* *,* *urban).*

The key recommendations included integrating comprehensive sexual and reproductive health (SRH) education into school curricula, training healthcare providers to offer respectful and empathetic care, and increasing accessibility through adolescent-friendly, school-based health facilities with free or subsidized services. Employing female healthcare providers and implementing peer education programs were also proposed to reduce stigma and foster supportive networks. Another participant highlighted the importance of school-based services, saying,

*"It would be better if a medical team came to school to conduct examinations and provide advice to all the girls, rather than waiting for them to go to the hospital."(Female, 15 years* *,* *urban).*

## Discussion

Our study shows an improvement in menstrual hygiene management practices among adolescent schoolgirls during the intervention period, largely due to increased access to hygienic menstrual products such as disposable and reusable pads and menstrual cups. This change was driven by the distribution of these products particularly reusable pads and menstrual cups as well as educational sessions that enhanced adolescents’ knowledge and awareness of proper menstrual hygiene practices. As a result, there was a change from unhygienic alternatives like cloths, fabric, and toilet paper, highlighting the positive impact of the intervention. These findings align with evidence from other low-income settings, where the combined approach of menstrual product distribution and menstrual health education has led to significant improvements in menstrual hygiene practices and overall health outcomes [30, 31].

The study highlights the effect of a comprehensive sexual and reproductive health intervention on the prevalence of RTIs symptoms among adolescent schoolgirls. The findings revealed both positive and positive trends. Symptoms such as thick, white, and clumped vaginal discharge, along with sores, blisters, or ulcers in the genital area, significantly decreased from baseline to endline. However, there was an increase in the prevalence of vaginal discharge with a strong, fishy smell, whereas the remaining RTIs symptoms did not change. In addition, variations in the prevalence of RTIs symptoms were observed across different age groups and places of residence, with older adolescents and rural residents experiencing improvements, whereas younger adolescents and urban residents faced worsening symptoms. The qualitative data revealed various barriers to adolescents accessing healthcare services for RTIs, including stigma, financial constraints, and inefficiencies within the healthcare system.

### Changes in the prevalence of reported RTI symptoms among adolescent schoolgirls

The study findings indicate that a comprehensive sexual and reproductive health intervention positively impacts the prevalence of certain RTIs symptoms. Specifically, there was a reduction in symptoms such as thick, white, and clumped vaginal discharge and sores, blisters, or ulcers in the genital area from baseline to endline. This improvement aligns with previous research showing that enhanced menstrual hygiene practices and increased access to health education and services can lead to a decrease in RTIs symptoms. Studies in Ethiopia and rural India have shown that promoting hygienic sanitary pad use and improving MHM practices significantly reduce RTIs [32, 33]. On the other hand, the study revealed a significant increase in the prevalence of vaginal discharge with a strong, fishy smell, increasing from 6% at baseline to 16% at the endline. This rise may indicate a growing issue related to bacterial vaginosis (BV), which is often characterized by such symptoms and is among the infections that can be related to menstrual hygiene. Similar trends have been reported in sub-Saharan Africa, where the incidence of BV has increased despite improvements in menstrual hygiene practices, possibly due to factors such as antibiotic resistance, changes in sexual behavior, or variations in the vaginal microbiota [34–38].

The stable prevalence of green or yellow vaginal discharge throughout the study period suggests that there was no significant change in the incidence of infections typically associated with these symptoms. The lack of change could be attributed to persistent gaps in access to appropriate diagnostic and treatment services or insufficient coverage of educational interventions addressing these specific infections. Other RTIs symptoms, including a burning sensation while urinating, general itching or burning in the genital area, sores, blisters, or ulcers, and rash or redness, showed minimal variation and lacked statistical significance. This indicates that while some symptoms might have been somewhat affected by the interventions during the study, the changes were not substantial. Similar findings have been reported in low-resource settings, where inadequate healthcare infrastructure, limited awareness, and cultural stigmas often constrain the effectiveness of public health interventions aimed at reducing health burdens, including RTIs, among women [39, 40].

The mixed results of the intervention reflect a complex web of factors, including sexual activity, immune response, and rectal microflora, which influence RTIs incidence by disrupting the vaginal ecosystem and contributing to infections beyond menstrual hygiene practices [41–44]. This underlines the need for multifaceted strategies that go beyond hygiene and health education to include broader social determinants of health, such as access to quality healthcare, comprehensive sexual health education, and effective diagnostic and treatment services. Future research should explore these factors more deeply to develop more effective interventions for reducing RTIs burdens among adolescent girls.

## Changes in the prevalence of reported RTI symptoms by age and place of residence (Urban/Rural)

The study revealed a slight impact of reproductive health interventions, with varying effects across demographic groups and contexts. Older adolescents and rural residents experienced significant improvements in RTI symptoms, such as reductions in vaginal discharge with a strong, fishy smell and burning sensations during urination. These positive changes align with existing research highlighting the success of age-targeted SRH interventions [45, 46]. On the other hand, younger adolescents and urban residents faced worsening symptoms, suggesting that interventions might need further refinement to address these groups'unique needs. Older adolescents typically possess greater cognitive maturity, which helps them better understand reproductive health issues. They have improved access to information and resources and often have more autonomy over their hygiene practices. Together, these factors lead to the adoption of healthier behaviors and more effective use of healthcare services. However, this study revealed a trend among younger adolescents, who experienced an increase in RTIs symptoms such as vaginal discharge with a strong, fishy smell and a burning sensation during urination. This suggests that existing interventions may not adequately address the unique needs of this age group. While improved menstrual hygiene education, access to menstrual products, and better WASH facilities are essential, they may not fully address hormonal changes and other puberty-related factors that can disrupt the vaginal flora and increase susceptibility to infections.

The study revealed that, compared with their rural counterparts, urban adolescents had increased RTIs symptoms despite better access to healthcare and hygiene facilities. This urban‒rural disparity suggests that factors such as healthcare quality, population density, and environmental conditions may contribute to the increased RTIs prevalence in urban areas. Owing to their relatively high levels of pollution and overcrowded conditions, urban environments may increase infection rates [47–51]. The findings support the idea that urban settings face unique reproductive health challenges and that interventions need to be specifically tailored to address these issues.

### Barriers faced by adolescent girls in accessing RTI treatment and strategies for improvement

Adolescent girls continue to face substantial barriers in accessing reproductive tract infection (RTIs) treatment, emphasizing the need for tailored interventions that enhance both healthcare access and utilization. The key challenges identified in this study systemic inefficiencies, financial constraints, sociocultural stigma, and inadequate support structures align with previous research [52–55], which emphasizes their significant influence on treatment-seeking behaviors and adherence to follow-up care.

Healthcare system barriers, including long wait times, medication shortages, and inconsistent follow-up care, remain critical issues, particularly in urban areas with high patient volumes [55–60]. These findings echo studies conducted in LMICs revealed that urban clinics, despite having more resources, are often overburdened and unable to meet the needs of adolescent patients promptly [61].

Financial constraints also severely limit access to RTIs treatment, particularly for rural populations, where additional challenges such as transportation costs further complicate access. This observation is consistent with other studies that identified cost as a major barrier in both rural and urban healthcare access, resulting in inadequate care [62–67]. The study also highlights the compounded difficulties faced by rural adolescent girls, noting that while financial and logistical barriers exist, the absence of adolescent-specific healthcare services exacerbates these challenges [68].

Sociocultural factors play a significant role in influencing healthcare-seeking behaviors. The stigma surrounding reproductive health, misinformation, and fear of judgment discourage many girls from seeking medical care. Previous studies have also highlighted how RTIs treatment is often erroneously associated with sexual activity, creating reluctance to disclose symptoms or seek medical attention. The fear of judgment and concerns over privacy contribute to the perpetuation of health inequities, as many girls avoid seeking help, even when symptoms persist [69, 70].

The results of this study underscore the need for adolescent-friendly healthcare models, as recommended by other previous studies [71, 72]. Strengthening healthcare infrastructure, integrating sexual and reproductive health (SRH) education into school curricula, improving healthcare provider training, and fostering community engagement are all critical strategies. School-based programs stand out as promising interventions, as they provide a platform to reach a wide audience of adolescents and reduce stigma through education and sensitization efforts. Research has demonstrated that school-based SRH programs can improve health knowledge, reduce stigma, and enhance healthcare-seeking behaviors [73, 74].

These findings highlight the urgent need for policy interventions that prioritize adolescent-friendly healthcare services. Addressing these barriers through comprehensive, multisectoral interventions is essential for empowering adolescent girls to manage their reproductive health more effectively. By reducing the prevalence of RTIs and improving overall health outcomes, these efforts can equip adolescent girls with the necessary knowledge, resources, and support to lead healthy and fulfilling lives.

### Strengths and limitations of the study

This secondary analysis uses pre collected data from the PASS MHW project, offering a cost-effective and efficient means to examine menstrual hygiene management, RTI symptom reporting, and barriers to healthcare access among school-aged adolescent girls. Although data were collected at two time points (baseline and endline), allowing for comparisons over time, the study employed repeated cross-sectional surveys rather than following the same individuals longitudinally. As such, while the design enables the identification of temporal trends and potential associations with the intervention, it limits the ability to draw causal inferences or assess individual-level changes in behavior or health outcomes.

Despite these design constraints, the study provides valuable insights into patterns of RTI symptom reporting, health-seeking behavior, and the broader contextual factors influencing adolescent reproductive health. The broad scope and large sample enhance generalizability, and the integration of follow-up qualitative interviews adds depth to understanding intervention pathways and system-level barriers. However, reliance on self-reported data may introduce recall and social desirability biases, and the original project's limited focus on RTI management constrains the specificity of some conclusions. Nonetheless, the findings generate actionable recommendations for strengthening health education, school-based screening, and referral systems, and highlight areas for future research using more robust longitudinal designs to establish causality.

## Conclusion

This study shows the effectiveness of a comprehensive sexual and reproductive health (CSRH) intervention in reducing self-reported RTI symptoms, particularly among older adolescents and those in rural areas. However, the increase in symptom reporting among younger adolescents may be due to improved awareness and recognition resulting from the intervention itself, highlighting the need for future studies to include objective clinical assessments or track symptom changes over time. The study also notes that not accounting for time since menarche may have influenced results, as menstrual experience not just age can shape responses to interventions. Despite these limitations, the findings underline the need for targeted strategies: younger adolescents require age-appropriate education to build knowledge and confidence, while urban adolescents face unique challenges that demand tailored, context-sensitive approaches. Enhancing healthcare access, school-based services, and equity-driven programming is crucial to reducing RTI prevalence and improving adolescent reproductive health outcomes.

## Data Availability

The datasets used/analysed for this manuscript are available from the corresponding author on request.
